# Nutritional aspects and dietary benefits of “Silkworms”: Current scenario and future outlook

**DOI:** 10.3389/fnut.2023.1121508

**Published:** 2023-01-19

**Authors:** Deepak Kumar Mahanta, J. Komal, Ipsita Samal, Tanmaya Kumar Bhoi, Vinod Kumar Dubey, Kiranamaya Pradhan, Aarthi Nekkanti, M. N. Rudra Gouda, Varun Saini, Nikita Negi, Sheenam Bhateja, Hansa Kumari Jat, Deepika Jeengar

**Affiliations:** ^1^Department of Entomology, Dr. Rajendra Prasad Central Agricultural University, Samastipur, Bihar, India; ^2^Department of Entomology, Navsari Agricultural University, Navsari, Gujarat, India; ^3^Department of Entomology, Sri Sri University, Cuttack, Odisha, India; ^4^Forest Protection Division, ICFRE – Arid Forest Research Institute (AFRI), Jodhpur, Rajasthan, India; ^5^Department of Entomology, University of Agricultural Sciences, Dharwad, India; ^6^Department of Entomology, Indira Gandhi Krishi Vishwavidyalaya, Raipur, India; ^7^Division of Entomology, Indian Agricultural Research Institute, New Delhi, India; ^8^Department of Seed Science and Technology, Chaudhary Charan Singh Haryana Agricultural University, Hisar, Haryana, India; ^9^Department of Entomology, Maharana Pratap University of Agriculture and Technology, Udaipur, Rajasthan, India; ^10^Department of Entomology, Rajasthan Agricultural Research Institute, Durgapur, Jaipur, Rajasthan, India; ^11^Department of Entomology, Govind Ballabh Pant University of Agriculture and Technology, Pantnagar, Uttarakhand, India

**Keywords:** nutrition, insect, silkworm, pupae, consumption, health

## Abstract

In the current scenario, it is estimated that by 2050, there will be an additional 2.5 billion people and a 70% increase in food demand. Crop yields are not increasing fast enough to support global needs, and world agriculture is facing several serious challenges. Therefore, insects can be a nutritious alternative to meet the ever-increasing food demand in the present and future. The majority of insect consumption occurs in developing countries, with approximately 1,900 insect species consumed worldwide. Food and feed derived from them are of high quality, have a high feed conversion ratio and emit a low level of greenhouse gases. Among insects silkworms are beneficial to humans, not only because of their high nutritional value, but also because of their several pharmacological properties. Silkworm eggs, larvae, and pupae contains high amount of proteins, oils, minerals, vitamins, and several other beneficial components which are nutritious as well as have positive effect on human health. Studies have shown that silkworm pupae protect the liver, enhance immunity, inhibit apoptosis, inhibit cancer, inhibit tumor growth, inhibit microbial growth, regulate blood glucose and blood lipids, and lower blood pressure. This review paper summerized the nutritional value of different life stages of silkworm, nutritional comparison of silkworm with the major human foods, and the effects of silkworm consumption on human health, thus ittargets to generate interest toward in sericulture and improve human health by using silkworm as a nutritious food and attain sustainability in food and nutritional security.

## 1. Introduction

The present world population has reached over 8 billion and is estimated to reach 10 billion by 2050. Due to a number of issues like land scarcity, unfavorable climate change, and insect and pathogen attacks, food production is slowly expanding in an arithmetic trend while the human population is swiftly rising in a geometric progression. Therefore, there will be a huge gap between the increasing population and food production, which is a major emerging problem in the near future. As demand for animal-derive protein is expected to increase at higher rate, insect based nutritional products can be marketed and commercialized as a new source of food. According to the FAO, at least 2 billion people already consume insects as part of their traditional diets. Currently, there are 2,111 edible insect species (1). Insects have historically been consumed by humans in Asia, Africa, and Latin America and have been the subject of extensive scientific study in recent years (2). Among the edible insects, silkworm larvae and pupae are one of the emerging insect-based nutritious food sources with high proteins, oils, polyphenols, vitamins, and chitosan (3). Silkworm eggs contain a high source of sugars, fat, and vitamin B (4). Various stages of silkworm larvae are used in pharmaceuticals (anti diabetic effect) or in food for supplementary nutrition (4). 18 amino acids and a high concentration of essential amino acids, which seem to be beneficial to humans, may be found in protein derived from silkworm pupae (5, 6). Unsaturated fatty acids, notably Omega-3 fatty acids, are abundant in silkworm pupae oil (7). The active compounds found in silkworm pupae have been shown in various experimental investigations to have significant favorable effects on human health. These advantages include antioxidant, anticancer, antibacterial, hepatoprotectant, and immunomodulator characteristics (8). Consequently, silkworms can be used in the near future in the biochemical, health, and food industries (9).

Silkworm is an endopterygote lepidopteran insect having complete metamorphosis with all the four life stages (egg, larva, pupa, and adult). The adults, after mating, lay eggs in batches (DFLs- Disease free layings), eggs, after hatching goes through five larval stages known as instars (I, II, III, IV, V). The larval stage of insect feeds on the host plant vigorously and produces a sericinous material called as silk thread. The late instar larvae produce cocoon by spinning sericigenous material around them called cocoon and pupa is produced inside the cocoon. There are different types of silkworms based on the host plant they feed on like mulberry silkworms, eri silkworms, tasar silkworms, and muga silkworms. Due to their great nutritional content and other medicinal properties, silkworm pupae are thought to be harvested for human consumption (10).

Further, sustainable development goal (SDG) 2 is about creating a world free of hunger by 2030. There were an estimated 720 million to 811 million hungry people in the globe in 2020, an increase of about 161 million over 2019. The number of people going hungry and suffering from food insecurity had been gradually rising between 2014 and the onset of the COVID-19 pandemic. The COVID-19 epidemic has worsened all types of malnutrition, especially among youngsters, and pushed already alarming rates even higher (11). Sustainable development in agriculture is based on increasing crop yields efficiently without putting undue stress on natural resources (12). The foundations of this farming approach, which also attempts to minimize the adversities on the environment, ensure long-term crop acquisition. Reduced chemical use and reliance on complicated machinery are only two examples of how sustainable agriculture strives to reduce its environmental impact (13). It is envisioned that environmental degradation can be avoided if agricultural producers’ commercial interests and customer quality demands are both respected. The circular economy concept is also worth considering in the context of sustainable development because it helps to reduce raw material consumption and trash generated during production by utilizing waste as substrates for subsequent activities (14). In this context, silkworms can be deployed as an alternative proteinaceous food source with lower greenhouse gas emission potential, helping to synergize the SDG target and alleviate the effects of climate change. Furthermore, the silk produced by larval instars, as well as the byproducts of the silk industry, can generate revenues in both impoverished and emerging economies.

This article’s goal is to examine the information on silkworm as a medicinal and food ingredient aligned with current SDGs. This review includes information from the disciplines of pharmacology, biochemistry, nutrition, and biomedicine. With the aim of elucidating the relationship between silkworm stages and health and offering a reference for their pharmaceutical use, the composition, functional mechanisms, and application prospects of silkworm stages (eggs, larvae, pupae, and adults) are explored.

## 2. Why silkworm as alternative food source and therapeutic option?

There are several reasons for which silkworm is considered a potential alternative of food source as well as therapeutic option. Global food demand is changing in terms of its composition as a result of rising wages and increased urbanization in developing nations, notably in Asia (8). The rise in meat consumption worldwide is mostly due to increased wealth (15). In 2030, high-income nations’ per capita meat consumption is anticipated to rise by 9%, Consequently, there will be a 48% increase in demand for coarse grain as livestock feed. The consumption of meat is closely tied to the need for grains and protein-rich diets (16). Animals are given around 6 kg of plant protein for every kg of high-quality animal protein generated (17). But, livestock production is a major contributor to global warming, with annual emissions estimated at 7.1 gigatonnes of CO_2_-equivalent, or 14.5% of human-induced GHG emissions (18). Therefore, achieving sustainability in the meat industry is a formidable task that calls for a multifaceted and holistic strategy, one that can be accomplished through the investigation of unconventional meat sources (19). By 2050, prices for beef, pigs, and poultry will be higher than they were in 2000 by more than 30% due to the rise in global prices for the most significant agricultural products (20). According to the same study, climate change may make matters worse and lead to an extra 18–21% price increase. When taking into consideration the increased demand for biofuels and declining agricultural production, food shortages may become even more serious: Land and labor productivity increased at a far slower rate (21). Future price increases in food and feed will lead people to look for alternate sources of protein, like silkworm.

About 18% of all greenhouse gas (GHG) emissions that are caused by humans worldwide come from the production of livestock, including the transportation of animals and feed (22). Enteric fermentation results in the production of methane (CH_4_), which is then released from manure (6% of world emissions); N_2_O is mostly emitted from fertilizer used for feed crops and manure (65% of global emissions). According to a survey of the literature, 1 kilograms of beef has the greatest environmental effect when expressed as CO_2_ equivalents (14.8 kg), followed by 1 kg each of pork (3.8 kg) and chicken (1.1 kg) (23). Over all of the world’s anthropogenic atmospheric ammonia emissions, which are to blame for eutrophicating surface waterways and acidifying soils, are produced by agriculture, with cattle contributing nearly two-thirds of this total (22). Ammonia and GHGs may both be produced by insects. In the hindguts of tropical species of termites (Isoptera), scarab beetles, and cockroaches (Blaberidae and Blattinae), methanogenic bacteria can be found (Scarabidae) (24). The majority of commercially raised edible insect species, such as the house cricket (*Acheta domesticus*), the migratory locust (*Locusta migratoria*), the yellow mealworm (*Tenebrio molitor*), and the silkworms do better than traditional cattle in terms of both direct emissions of GHG and ammonia generation (25).

Since a rise in the demand for meat would result in a greater-than-proportional rise in the need for grain and high-protein feeds, feed conversion ratios (FCRs) are particularly crucial. Depending on the type of animal and the methods used to create the meat, FCRs might vary greatly. A very basic computation may be conducted, nevertheless, using average data. The following FCRs were determined using long-term data for the United States: 2.5 for poultry, 5 for pork, and 10 for beef (26). From this study, it is estimated that the FCR for silkworm larva is nearly 7.5, whereas for pupa is 16 (27). Traditional animals and insects have quite different weight ratios that make them edible. The percentage of edible weight is nearly 90%, which is far more than chicken (55%) (28) and beef (40%) (26).

The prevalence of livestock diseases and the emergence of novel, frequently antibiotic-resistant diseases are two additional effects of high-density animal production systems. The worldwide population loses billions of dollars each year due to infectious illnesses that affect cattle, such as foot-and-mouth disease, bovine spongiform encephalopathy (BSE), avian influenza (H5N1), and classical swine fever (29). Additionally, concerns with human health like BSE (29) and cardiovascular disease and cancer have been linked to meat eating in high-income nations (30). Zoonotic illnesses are on the rise and pose serious risks to human health, including the new strain of influenza A (H1N1), which is closely linked to swine influenza A (31). These dangers are anticipated to be quite modest given that insects (silkworms) are taxonomically far more distant from humans than conventional cattle.

Significant worldwide virtual water flows are associated with trading in cattle and livestock products (nearly half of the volume of virtual water flows relates to feed crops) (32). This is due to the fact that animal products, especially livestock with its 22,000 liters kg^–1^ produced, have a relatively high virtual water content compared to cereal crops. Due to indirect water inputs like forage and grain feed crops, other sources even claim 43,000 liters (33). A larger amount of meat in the diet causes water scarcity. The numbers for certain edible insect species like silkworm are anticipated to be significantly lower.

## 3. Silkworm eggs

**FIGURE 1:**
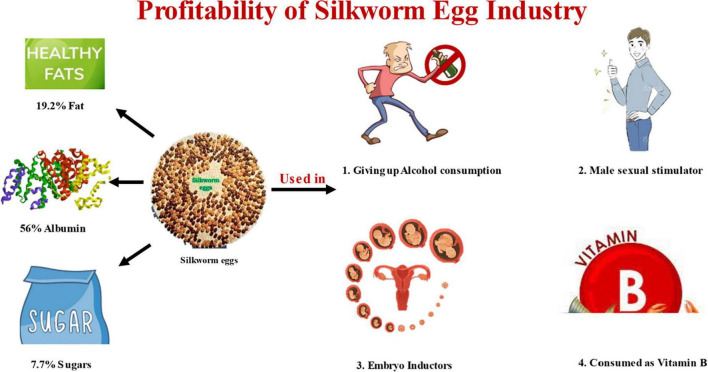
Representing amount of fat (19.2%), albumin (56%), and sugars (7.7%) in silkworm egg. The figure is also depicting about different functions of silkworm eggs like giving up alcohol consumption, male sexual stimulator, embryo inductor, and source of vitamin B.

The proteomic studies discovered many proteins, including proteins for storing food in silkworm eggs (34). The silkworm eggs contain three main types of glycoproteins namely vitellin, 30 kDa protein, and egg-specific protein (ESP) which account for more than 90% of total (35). Silkworms produce numerous 30 kDa low-molecular-weight proteins, which account for 35% of the total soluble protein in the egg (36). More than thirty genes coding for 30 kDa proteins have been annotated from *Bombyx* genome. Several additional proteins, including enzymes, proteases, and inhibitors of proteases, were also discovered in the eggs, along with parts of the silkworm energy metabolic system. Enzyme cathepsin B-like acid cysteine proteinase in the eggs of silkworm moth, *Antheraea pernyi* identified which help in the process of embryogenesis (37). A silkworm egg contains 56% albumin, 19.2% fat, and 7.7% sugar (4). The silkworm eggs contain high amount of protein and vitamins B1, B2. In Romania food industry silkworm eggs are used as Human fort B product (4). Silkworm eggs can be a potential nutritious food for nursing mothers due to presence of omega-3 polyunsaturated fatty acids. This unsaturated fatty acids are precursors of prostaglandins which has significant role in infants nutrition. Consumption of silkworm eggs increases the male sexual power and decreases erectile dysfunction as it increases GSH and nitric oxide synthetase levels in the corpus cavernosum. Silkworm eggs are said to cause heavy drinkers to stop drinking alcohol completely if they eat them (Popular tradition, India). Silkworm eggs are used as embryo inducer, hepatic protector, hypolipidic, and hypoglycemic. The silkworm eggs are also used extensively in transgenic studies (38). The nutritional composition and medicinal uses are mentioned in Figure 1.

## 4. Silkworm larvae

**FIGURE 2:**
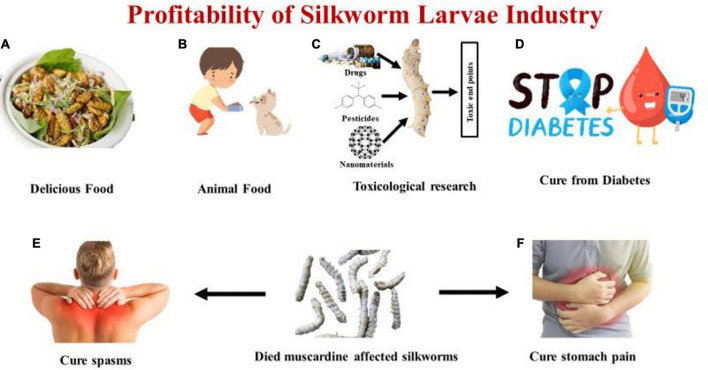
Representing **(A)** Delicious food prepared from silkworm larvae. **(B)** Silkworm larvae used as animal food. **(C)** Silkworm larvae used as toxicological research studies for drugs, pesticides, nanomaterials. **(D)** Silkworm larvae used for curing blood sugar level (Diabetes). **(E)** Died fungus affected silkworm larvae curing body spasms. **(F)** Died fungus affected silkworm larvae curing stomach pain.

It is estimated that 100 grams of the larvae of *Bombyx mori* consist of 54% protein, 8% fat, 6% fiber, and 6% ash, as well as 390 kcal of energy (39). Due to their high protein content, larvae are fed to younger animals, including reptiles, as a nutritional supplement. Pharmaceutical companies use a complete proteic extract from silkworm larvae possessing anti-diabetic activity, or the food industry uses it an additional nutraceutical (40). According to the research, the silkworm has the greatest potential to drop blood sugar when it is prepared on the third day of the fifth instar, created using the freezing-dry method, and taken as powder as compared to other methods (41). Due to their significant hypoglycemic activity, which is caused by 1-deoxynojirimycin (DNJ), which they obtain by feeding mulberry leaves, silkworms are attracting attention from all over the world. Silkworm DNJ content fluctuates dramatically during its life cycle as a holometabolic insect. DNJ content was analyzed by reverse-phase high-performance liquid chromatography (RP-HPLC) and it was estimated that 3rd day of V instar larvae possesses the highest level of DNJ (1512.46 μg per silkworm) (42). Silkworm has traditionally been used as a diabetic treatment in oriental nations such as China, Korea, and Japan, and several studies have recently proven the silkworm’s blood glucose-lowering impact. Humans can readily digest and absorb silkworm powder. It can also improve the physiological activities of the gastrointestinal tract (43). Cramps, bloating, and other illnesses can be treated using dehydrated silkworm larvae that were killed by the white muscardine disease (44). It is believed that mulberry-eating silkworms have better health benefits than other species. Due to their susceptibility to chemical substances like pesticides, medicines, and heavy metals, silkworms (*Bombyx mori*) are an excellent model organisms in health safety and ecological pollution evaluation (45). Different profits of the silkworm larva industry are mentioned in Figure 2.

## 5. Silkworm pupae compositions

Silkworm pupae contain a wide range of nutrients. The most prevalent constituents are protein, fat, and sugar, as well as minerals, vitamins, polyphenolic compounds, and a variety of other nutrients (46, 47). We have reviewed and summarized them individually here.

### 5.1. Proteins in silkworm pupae

**FIGURE 3:**
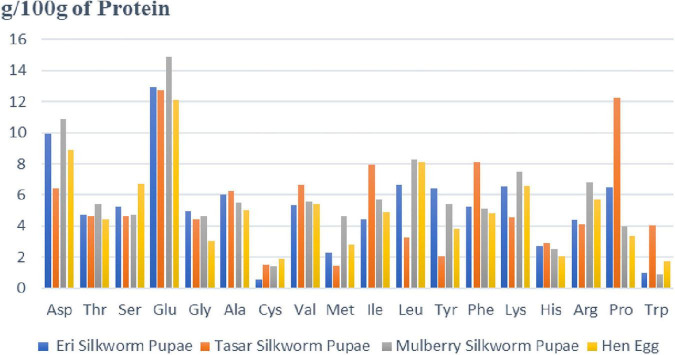
Representing amount of different amino acids (g amino acid/100 gm of protein) present in different silkworm pupae as compared with hen egg. The figure depicts that amount of essential and non-essential amino acids present in silkworm pupae are higher than the egg of hen. The amount of amino acids present in mulberry pupae is far more than the other silkworm pupae as well as egg of hen. The amino acid composition of the proteins is essentially the same in the different species of silkworm pupae, all consisting of 18 amino acids (except for Eri silkworm pupae). Compared to hen eggs, pupae are higher in Phe and Pro.

*Bombyx mori* has the highest protein content in silkworm pupae (55.6% dry weight) and is the most prevalent dry matter (48). The biological active peptides, made up of more than a dozen amino acids are involved in several physiological functions (49). The proteins present in silkworm pupae get hydrolyzed and converted into several biologically active compounds that are used to perform the pharmacological functions of the silkworm pupae. The amino acid makeup of the proteins in different species of silkworm pupae is roughly the same, with all of them consisting of 18 amino acids. Eight of these essential amino acids fulfill the WHO/FAO/UNU recommendations. There are another ten non-essential amino acids that are required by humans. Phenylalanine and proline levels in silkworm pupae are greater than in hen eggs (50). Figure 3 represents different amino acids (g amino acid/100 g of Protein) present in different silkworms (Eri, Mulberry, and Tasar) in comparison with nutritious food that is egg of hen (3, 48). It is shown that maximum of amino acids are present in more quantity in comparison with egg. Amino acids like asparagine, threonine, glutamine, glycine, alanine, methionine, isoleucine, leucine, lysine, proline are more abundant in silkworm eggs as compared to hen eggs. Figure 3 indicates that mulberry silkworm pupae are rich in most of the amino acids as compared to other silkworms and eggs of hens (46, 47, 50).

### 5.2. Minerals in silkworm pupae

**FIGURE 4:**
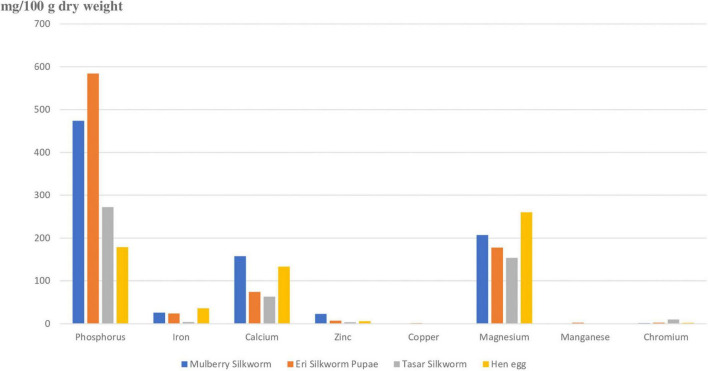
Depicting amount of minerals present in pupae of different types of silkworms against minerals present in egg. Phosphorus, magnesium, and calcium are the major minerals present in silkworms. Silkworm pupae have more minerals as compared to hen egg. Phosphorus is highest in eri silkworm, Calcium, and Zinc is highest in mulberry silkworm and chromium in tasar silkworm. The amount of iron is very less in tasar silkworm. Manganese is present very less amount in all the silkworms and almost absent in tasar silkworm, Among all the minerals present in silkworms phosphorus is present in highest amount. The amount of chromium is present least in eri silkworm.

There are nearly 25 different types of minerals, each of which plays a different key role in different physiological functions in organisms (51, 52). Figure 4 represents eight major minerals present in silkworm pupae (3). Figure 4 depicts that phosphorus, magnesium and calcium are present in greater amounts in pupae (mg/100g dry weight). The content and type of mineral present in pupae depends upon the type of silkworm pupae (Eri, mulberry, tasar), environment and host plant they feed upon (50). It is shown that the Na:K (sodium to potassium) is very low in silkworm pupae. As presence of Na and K increases the occurrence of non-communicable diseases, consumption of silkworm pupae will reduce spreading of non-communicable diseases like stroke, hypertension, and cardiovascular disease (53, 54). Silkworm pupae are rich in minerals like phosphorus, iron, calcium, zinc, manganese, and chromium as compared to hens’ eggs. Phosphorus is highest in pupae of Eri silkworm whereas mulberry silkworm is rich in calcium and zinc. Tasar silkworm pupae are rich in chromium.

### 5.3. Oil in silkworm pupae

**FIGURE 5:**
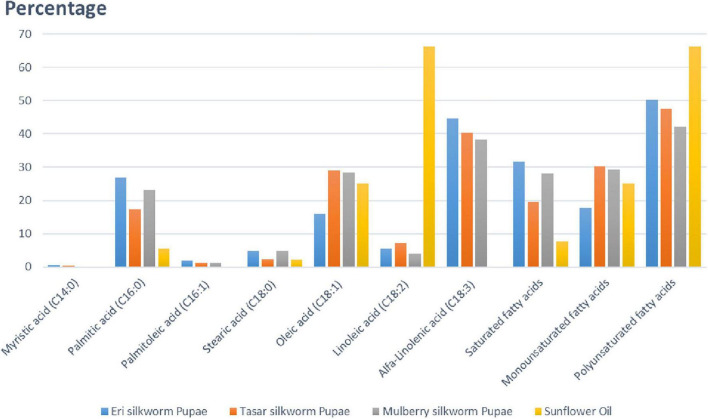
Representing quantity of fatty acids (in percentage) present in pupae of silkworms (mulberry, eri, tasar) in comparison with sunflower oil. Palmitic acid is more in eri silkworm pupae, Oleic acid is more in tasar silkworm, Saturated fatty acids are more in eri silkworms, polyunsaturated fatty acid is more in eri silkworm pupae in comparison with other silkworm pupae. Out of four different species of silkworm pupae, Eri silkworm pupae has the highest oil content, at 26.2%. All the different silkworm pupae oils contain high levels of unsaturated fatty acids (both monosaturated and polysaturated), with 77.71% in *Antheraea pernyi*. Silkworm pupae are not only rich in oils but also contain high levels of unsaturated fatty acids, especially polyunsaturated fatty acids.

Oil is the second most abundant component in silkworm pupae after protein. Among the four species of silkworm pupae, eri silkworm pupae have the highest oil content, at 26.2% (47). Different saturated and unsaturated fatty acids (in percentage) present in pupae of different silkworms (mulberry, eri, and tasar) in comparison with sunflower oil are summarized in Figure 5. Based on the figure, tasar silkworm pupae oil contains the highest amount of unsaturated fatty acids among all silkworm pupae oils. Additionally, silkworm pupae contain the omega-3 fatty acids eicosapentaenoic acid and docosahexaenoic acid, both of which promote human health (55). Silkworm pupae contain not only high levels of oils but also highly unsaturated fatty acids, particularly polyunsaturated fatty acids, which are important sources of edible oils (7). Palmitic acid is more in eri silkworm pupae, Oleic acid is more in tasar silkworm, Saturated fatty acids are more in in eri silkworms, polyunsaturated fatty acid is more in eri silkworm pupae in comparison with other silkworm pupae (Figure 5).

### 5.4. Other silkworm pupae ingredients

Silkworm pupae are also rich in vitamins and contain a variety of these nutrients. Vitamins found in silkworm pupae include Vitamin A, B1, B2, B3, B5, B7, B9, B12, C, and E (56). Phospholipids and five tocopherols, which include α-tocopherol, β-tocopherol, γ-tocopherol, γ-tocotrienol, and σ-tocopherol are present in silkworm pupae (57). A concentration of 10 mg/g of polyphenols and 20 mg/g of flavonoids was detected in the pupae of the silkworm *Antheraea assamensis* (58). All of the sugars found in silkworm pupae are biologically active, including chitosan, chitin, and isolated polysaccharides (3, 59). Carboxymethyl chitosan, especially from silkworm pupae, has strong physiological activities but is not cytotoxic (60). Silkworm pupae also contain rare dimethyladenosine derivatives (61). As a result of these biofunctional activities, silkworm pupae have certain pharmacological functions.

### 5.5. Silkworm pupae as a nutritious and delicious human food

Pupa oil contains attributes including an acid score of 67.37, 1.47 index of refraction at 30^°^C, an iodine value of 174.91, a saponification value of 150.88 and with 0.36 percentage of cholesterol, making it a strong fit for exploitation in the food industry (62). As a delectable human food, silkworm pupae are used in various Asian nations like Thailand, Korea, India, China, and Japan. Silkworm pupae have been discovered to boost lactating ability in tribal women and are superior to soya beans, salmon, or beef in terms of protein content. They are also comparable to meat in terms of calories, fat, protein, and vitamins (4, 63–65). Pupae have a large amount of crunchy chitin in their exoskeletons, which can be a useful supplement to rural populations’ cereal diets. Pupae have a lot of commercial potential when used in chili sauce or chocolate. Pectin, a byproduct of silkworm pupae, was used to thicken ice cream, jam, candy, jelly, and fruit juices (63). Chitin, a component of pupal skin, is used for a variety of purposes, notably as an ingredient to increase loaf volume in wheat flour bread, and silkworm pupal cakes are produced and marketed in Japan due to their high nutritional content (66). In China, Japan, Hong Kong, and Korea healthy silkworm pupae are sterilized, vacuum-dried, and sold as commercial food products, whereas cocoon Palade powder was utilized in sauces and soups (67). The eri pre-pupae and pupae are used to make the delicious fry, pakori, chop, and cakes in India (64). Shinki fibroin, a hydrolyzed byproduct of discarded silk fiber, is consumed with milk or coffee, while the free amino acids extracted from the cocoon palade are widely employed in the food sector as a low-cost raw material (68). In Africa, raw, dehydrated, and ground saturniid (silkworm) larvae serve as a garnish, while in the Western United States, the peaggie, which is a dried form of pupae, is consumed as food. Silk protein can be used to make a variety of foods and drinks (69) and might be incorporated into the diet of the crew of Control Ecological Life Support (CELIS), one of the most intricate and complicated closed ecological systems in the world (70). The most pressing issues are the dietary constraints in accommodations for extended stays. Recent research has proved that silkworm meal can meet astronauts’ nutritional needs for long-term space missions due to its great quality of protein content, proper quantities of amino acids, unsaturated fatty acids, and other necessary dietary components for humans (71). During the 36th Scientific Assembly of the Committee on Space Research (COSPAR), the Japan Aerospace Exploration Agency (JAXA) announced a pupal recipe as astronaut food (72).

### 5.6. Silkworm pupae as a nutritious animal feed

Silkworm waste pupae or SWP, are a rich source of nutrients for livestock and poultry. Among many other protein sources, SWP are regarded as a crucial dietary protein supplement for poultry with sufficient processing at an affordable price (73). Silk waste and pupae are used in fish and poultry feed (73). Hens fed with de-oiled pupae showed a greater tendency of increased egg production and improved egg yolk color, while fat-free pupae were fed to fish and carps to increase yields (74). The cost of dried pupae increased to INR 13–15 per kg from INR 2–3 due to the hybrid magur fish’s greatly improved growth, which brought in 4–5 times more profit (4). The dried pupal diet improved egg quality and growth rates in hens while improving survival, feed conversion, and specific growth rates in fish. Diets high in deoiled pupae led to weight gain and fur growth in rabbits (72).

### 5.7. Silkworm pupae in cosmetic and chemical industry

**FIGURE 6:**
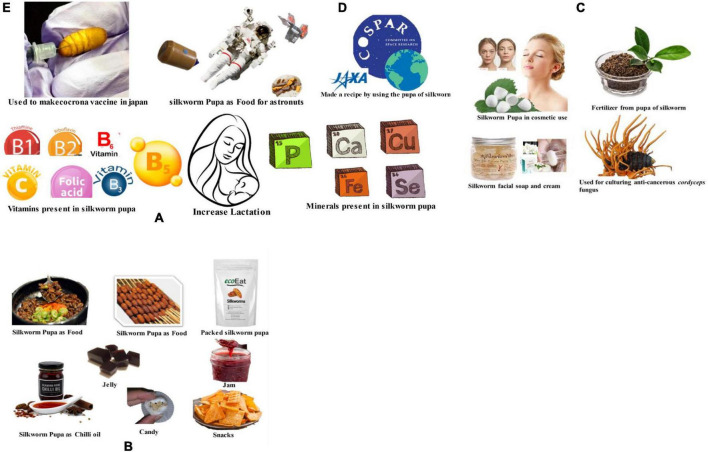
Representing benefits of silkworm pupae industry **(A)** silkworm pupae contains several vitamins and important minerals those helps in increasing lactation for mother. **(B)** Representing silkworm pupae in food industry which is available in several forms like delicious cooked food, packed silkworm pupa, chili oil, candy, jelly, jam, snacks. **(C)** Silkworm used in cosmetics and chemical industry producing soap, cream, fertilizer. It is also used as culturing agent for cordyceps fungus (anti-cancerous). **(D)** Silkworm pupae used as food supplements in space for astronuts due to high protein content. **(E)** Silkworm pupae used for making vaccine for corona vaccine.

Silkworm pupae, which have a high amount of fat (approximately 30%), are used to produce chrysalis oil, which is utilized to produce cosmetic products (emulsions, soaps, creams, and lotion). Examples of such materials include the protein derivative of the pupal skin, chitin used in cosmetics, and absorbent/resilient hybrid silk films used in scar de-scarring and wound healing (70, 75). Silkworm pupal fat and oil, which has been shown to slow the signs of aging, darken gray hair, and help people lose weight, can benefit the soap and cosmetics industries. The silkworm pupal oil is utilized in cosmetic products including body deodorants, face powder, and hair oils (72). The food processing and oleochemical industries utilize silkworm pupae oil extensively. The silkworm contains significant amounts of n-triacontanol, a substance used to boost plant growth. Pupal skin, frequently available as waste in the reeling and grainage industries, can be used as a marketable raw material for many different industries (75, 76). Because of this, silkworm pupae are utilized in a variety of sectors, including the culinary, cosmetics, pharmaceutical, and chemical industries (Figure 6).

### 5.8. Silkworm pupae: Pharmacological mechanisms and functions

**FIGURE 7:**
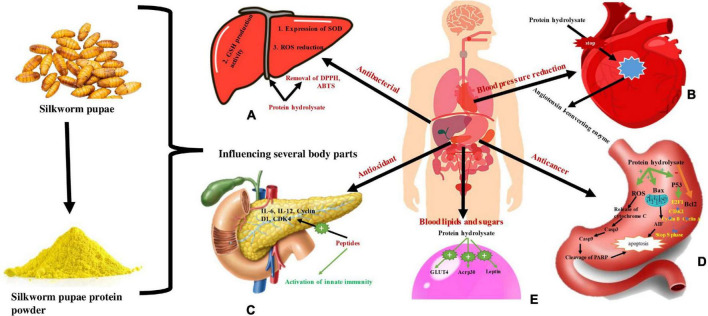
Representing use of silkworm pupae proteins in different function and physiological mechanisms. **(A)** Consumption of pupal protein is used as antibacterial agent as protein hydrlysate regulates GSH production activity, Expression of SOD and ROS reduction. **(B)** Pupal protein is used for reducing blood pressure by inhibiting Angiotensin I-converting enzyme by protein hydrolysate. **(C)** Pupal protein is used as antioxidant as it activate innate immunity and activate IL-6, IL-12, Cyclin, D1, CDK4. **(D)** Pupal protein is used as anticancer agent as protein hydrolysate activates ROS, Bax, P53 which cause apoptosis and also stop S- phase of cell division. **(E)** Pupal protein impact on decreasing blood lipids and sugar level as protein hydrolysate activate GLUT4, Acrp30, and Leptin.

Silkworm pupae’s active ingredients have a variety of pharmacological uses and powerful therapeutic effects for a variety of diseases. Studies done *in vivo* and *in vitro* have shown that silkworm pupae have strong pharmacological effects. Examples of these effects include their antibacterial, antitumor, antiapoptotic, antioxidant, hypotensive, immunomodulatory, lipid- and blood sugar-regulating, and hepatoprotective activities (Table 1 and Figure 7).

**TABLE 1:** Representing pharmacological functions of different species of silkworms, functional ingredients present in pupae and mechanisms involved with different pharmacological functions.

Species of silkworm pupae	Pharmacological functions	Functional ingredients	Occurring mechanism or effect	References
*Antheraea assamensis*	Antioxidant	Methanolic pupae extract	Free-radical scavenging activity that is dose-dependent.	(58)
		Polyphenols	High ROS scavenging activity was observed. Effective as a natural antioxidant in the development of protein-rich foods.	(135)
*Antheraea mylitta*	Antioxidant	Polyphenols	High ROS scavenging activity was observed. Effective as a natural antioxidant in the development of protein-rich foods.	(135)
*Bombyx mori*	Antitumor	Protein hydrolysates	Shows potent anticancer activity by inhibiting the proliferation of human gastric cancer cells SGC-7901.	(84)
		Protein hydrolysates	Structural changes in intracellular organelles of MGC-803 gastric cancer cells leading to vacuolization and rupture.	(86)
		Protein extracts	Significantly reduces the IL-6, IL-1, TNF- levels, protein, and nucleic acid content of breast cancer cells MCF-7.	(85)
	Antioxidant	Protein hydrolysates	Promotes ABTS free-radical scavenging activity thereby preventing diseases caused by oxidative stress.	(77)
		Protein hydrolysates	In Hepatic HepG2 cells, higher activity of ROS reduction and superoxide dismutase expression.	(136)
		Polyphenols	High ROS scavenging activity was observed.	(135)
	Antibacterial	Chitin and chitosan	Antifungal activity is superior to commercially available chitosan. Also, it can be utilized as a sustainable source of chitosan.	(91)
		Peptides	The Peptide Ranker and the CAMP database found peptide sequences with potential bioactivity with the highest score.	(137)
		Oil	Effective antibacterial activity against the gram-positive *Bacillus subtilis* and *Staphylococcus aureus*.	(88)
	Antiapoptotic	Silkworm Protein 30Kc6	30Kc6 prevented oxidized low-density lipoprotein-induced cell death in HUVEC cells by blocking MAPK signaling pathways aiding in human cardiovascular disease prevention and treatment.	(103)
		Silkworm haemolymph	The silkworm’s haemolymph may directly affect the baculovirus-induced apoptosis cascade or promote the expression of antiapoptotic baculovirus genes such as p35 in insect cells (Sf 9) infected with baculovirus (AcNPV).	(96)
		Recombinant 30 K protein	In humans (HeLa cells) and insect cells (Sf 9), recombinant 30 K protein prevents apoptosis triggered by viruses or chemicals.	(97)
		Silkworm haemolymph	Silkworm haemolymph inhibited apoptosis, which reduced cell detachment from an adhering surface. Thereby being helpful in preventing cell death.	(99)
	Blood pressure reduction	Protein hydrolysates	Angiotensin I-converting enzyme inhibitory action is found in silkworm protein hydrolysates.	(92)
		Protein hydrolysates	Angiotensin-converting enzyme was inhibited in a competitive manner. The mode of angiotensin-converting enzyme inhibition was competitive.	(138)
		Protein hydrolysates	By flexible docking calculation, the peptide inhibitory activity was 0.047 mg/ml in IC50, and it was bound to Asp415, Asp453, Thr282, His 353, and Glu162 in the hydrogen bond to the angiotensin-converting enzyme active pocket.	(93)
	Antiageing	Oils and sericin	Oils and sericin have tyrosinase inhibitory and free-radical scavenging properties *in vitro*. Hence used in cosmetics for whitening.	(109)
	Alcohol detoxification	Extracts	There is a significant increase in alcohol dehydrogenase activity in the livers of mice suggesting that the extract could be employed as a therapeutic substance to help people avoid hangovers.	(106)
	Anti-Alzheimer’s disease	Silkworm pupa vaccine	Recombinant proteins synthesized in domestic silkworm pupae improved memory and cognitive performance in mice. Hence, very nutritious CTB-A15 silkworm pupae vaccine could be used to prevent Alzheimer’s disease in the future.	(139)
		Silkworm pupae Powder	Hippocampal memory deficit was significantly reduced *in vivo*, as was hippocampal neuron density. Silkworm pupae appear to be a potentially useful meal for Alzheimer’s disease prevention.	(140)
	Antifatigue	Powders of silkworm, pupae, dongchung hacho, and silk powder	Increases swimming time and muscle mass in mice while reducing tiredness. It has anti-fatigue properties and may help athletes perform better.	(108)
	As bioreactor		Silkworm nucleopolyhedrosis virus was used to successfully express the human granulocyte-macrophage colony-stimulating factor in silkworm pupae. For heterologous protein expression, the silkworm pupa is a convenient and low-cost bioreactor.	(110)
	Immune regulation	Polysaccharide	Innate immunity is turned on in penaeid prawns, it effectively inhibits vibriosis.	(141)
		Peptides	Immune-related factors such as interleukin-6 and interleukin-12, nuclear factor-B, cyclin D1, and cyclin-dependent kinase 4 are all stimulated. Hence it has an immunomodulatory function and may have medicinal promise.	(142)
	Blood lipid reduction; weight loss	Peptides	Adipogenesis is inhibited resulting in a decrease in body weight gain. Alternatives to reduce dietary obesity that have no negative effects could be viable options.	(143)
	Blood glucose regulation	Soluble fibroin	In 3T3-L1 adipocytes, fibronectin promotes glucose absorption and metabolism. This could explain why the body’s response to fiber improves diabetic hyperglycemia.	(144)
		Purified fibroin	The findings show a decrease in glucose levels in the haemolymph. Diabetes, obesity, and other lifestyle-related disorders may benefit from this supplement.	(95)

#### 5.8.1. Silkworm pupae as antioxidant

Many polyphenols and peptides with antioxidant properties are now found in silkworm pupae and extracted using a variety of methods. These substances are effective at scavenging ABTS (2,2′-azino-bis(3-ethylbenzothiazoline-6-sulfonic acid) and DPPH (2,2-diphenyl-1-picrylhydrazyl) free radicals as well as intracellularly generated ROS (Reactive oxygen species), according to *in vitro* tests (77–79). According to studies (78) two peptides from silkworm pupae have significant antioxidant function in HepG2 cells as evidenced by superoxide dismutase expression, ROS reduction and glutathione (GSH) production. In DPPH and ABTS radical scavenging tests, a 30% ethanol extract of pupae was discovered to possess the highest antioxidant activity. Additionally, it was shown that the antioxidant activity in pupae varied by sex and age, with female pupae in the early stages of pupation showing greater free radical and ROS scavenging (79). According to a study on using the technique of extraction of pupa oil by microwave-assisted retrieval, there was an increase in the yield of silkworm pupa oil and also resulted higher overall phenolic content in the oil, which ultimately encourages a stronger antioxidant potential than that attained with the conventional extraction method (80). It may be possible to produce foods or pharmaceuticals that have antioxidant properties because of the peptides, phenols, and unsaturated fatty acids in silkworm pupae. Due to their hydrophilic nature, permeability, and multimodal interactions with the biological environment, natural antioxidants play vital roles for the prevention of diseases. On the other hand, synthetic antioxidants may obstruct the biological actions of other nutrients in the body. Natural antioxidants are therefore generally perceived as being safer than synthetic antioxidants (81, 82). These compounds are antioxidants in nature therefore, the outcome might be enhanced and rendered safer.

#### 5.8.2. Silkworm pupae as anticancer agent

Discovering natural antitumor therapies might be a better cancer treatment option than the radiation and chemotherapy now employed. *In vitro* studies have shown that the amino acids and protein hydrolysates in pupae have anticancer capabilities and are lethal to the cancer cells of the human breast, stomach, and liver (83–85). The anticancer properties of silkworm pupa oil and protein have both been demonstrated. Silkworm pupae proteins influence the cancer cells’ cycle of cell division and trigger the synthesis of apoptotic factors, which aid in apoptosis, acting as anticancer agents. Cancer cell mitochondria are affected by silkworm pupae protein, which in turn impairs the operation of their energy metabolism and triggers the apoptotic flux, which kills the cancer cells (86). In contrast to the anticancer effects of silkworm pupae proteins, the mode of action of the amino acids in pupa oil is through the production of ROS in cancer cells, which ultimately prevents liver cancer through cell apoptosis (83). It might be possible to use silkworm pupae as a source of anticancer medications if further investigation into them is performed.

#### 5.8.3. Silkworm pupae as anti-bacterial agent

Despite the lengthy history of usage of silkworm pupae in medicine, the understanding of their antibacterial properties has only lately evolved. The growth of a *Staphylococcus sciuri* strain, CD97 was strongly suppressed by silkworm pupa oil when its antibacterial activity was tested using the minimum inhibitory concentration (MIC) technique; the best results were obtained at 110 L/ml (87). Additionally, it was shown that Gram-positive bacteria were more susceptible to the antibacterial effects of hot-pressed, extracted silkworm pupa oil (88). Chitin and chitosan, which are abundant in silkworm pupae shells and have high antibacterial characteristics, are employed in a variety of biomedical applications (89, 90). Chitosan in silkworm pupae was discovered to be 67% acetylated and 48% crystalline. The antibacterial and antifungal efficacy of silkworm pupae-derived chitosan was superior to that of commercially available chitosan, with the quickest bacterial suppression occurring at 1–2 h (91). Silkworm pupae contain an antibacterial substance that could be utilized to treat illnesses and cut down on the excessive use of antibiotics.

#### 5.8.4. Silkworm pupae as blood lipid, blood pressure, and blood sugar regulator

Angiotensin-converting enzyme activity is increased in hypertensive individuals, but silkworm pupae protein hydrolysate greatly reduces angiotensin-converting enzyme activity. At a concentration of 2.0 mg/ml of silkworm pupa protein hydrolysate, the angiotensin-converting enzyme was inhibited by 73.5%, whereas the optimized hydrolysis procedure produced a semi-inhibitory concentration of 1.4 mg/ml (92). Additionally, a peptide derived from the protein of silkworm pupae exhibited a potent inhibitory impact on the activity of the angiotensin-converting enzyme, with a semi-inhibitory dose of 0.047 mg/ml (93). Rats that were naturally hypertensive were fed various concentrations of silkworm pupae protein hydrolysate for 4 weeks. It was discovered that the rats’ systolic blood pressure reduced, and that the decline depended on the concentration of the hydrolysate (94). Since silkworm pupae protein hydrolysate has demonstrated a hypotensive effect *in vitro* and *in vivo*, it may be produced and used as an antihypertensive medication or a dietary supplement to treat hypertension. Blood sugar and blood lipid levels are also regulated by silkworm pupae. Alpha-glucosidase is inhibited by silkworm pupae powder, which also decreases post-prandial blood sugar levels. Additionally, it increases fat metabolism and decreases fat deposition in rats (95). This indicates that silkworm pupae have the potential to be developed into a medicine to decrease blood sugar levels in diabetics while also having the same impact on weight reduction.

#### 5.8.5. Silkworm pupae as antiapoptotic agent

A low-molecular-weight lipoprotein abundant in silkworm pupae has been identified as a member of the 30 K family of lipid-transporting proteins that also inhibits apoptosis in mammalian cells (96–98). Haemolymph from silkworm pupae was found to suppress apoptosis in virus-infected insect cells and increase the longevity of the cells. Then, a monomeric protein with antiapoptotic characteristics that wasn’t glycosylated was found in the hemolymph (96). Later research revealed that a 30 K protein was the antiapoptotic agent, and in *E. coli*, a recombinant 30 K protein was produced that, like haemolymph, prevented viral or artificially triggered apoptosis (97). Further investigation revealed that silkworm pupae haemolymph similarly prevented human cell apoptosis from occurring (99). Significant gains for *in vitro* cell culture result from these discoveries.

#### 5.8.6. Silkworm pupae as hepatoprotective agent

Studies on animals have revealed a preventive effect of silkworm pupae oil against stomach ulcers caused by ethanol and hydrochloric acid. Silkworm pupae oil increased the pH of the stomach in mice and reduced the size of peptic ulcers and gastric discharge. In rats with gastrointestinal ulcers, silkworm pupa’s oil decreased serum levels of IL-6, IL-12, TNF-, IFN-, MTL, and GT while increasing levels of SST, SOD, GSH-Px, VIP, and CAT. While eNOS, EGF, EGFR, and VEGF expression were up-regulated, COX-2, iNOS, NF-B, and Bcl-2 expression were down-regulated. This data demonstrates that applying silkworm pupae oil to mice reduces inflammation and oxidative stress (100). Silkworm pupae oil also reduced the acute liver damage and oxidative stress brought on by alcohol, acetaminophen, and other drugs in rats by inhibiting the oxidative stress mediated NF-B signaling pathway (101, 102). Potential medication sources include silkworm pupae, which may be used to treat stomach ulcers and prevent acute liver damage in humans in the future.

#### 5.8.7. Silkworm pupae as cardiovascular protective agent

Research has shown that the silkworm pupae’s 30 K protein has antiapoptotic activity in addition to having cardiovascular disease prevention properties. The 30Kc6 protein from silkworm pupae reduced the severity of aortic and liver lesions as well as serum levels of low-density lipoprotein cholesterol (LDLC), high-density lipoprotein cholesterol (HDL-C), total triglycerides (TG), and total cholesterol (TC) in atherosclerotic rabbits (103). Additionally, it has been shown that raw silkworm pupae extracts improve the health of atherosclerotic rabbits, most likely due to their antioxidant and hypolipidemic actions (104). It has been discovered that the sodium salt of silkworm pupae oil considerably lessens the aberrant migration and proliferation of vascular smooth muscle cells brought on by platelet-derived growth factors. Vascular smooth muscle cells treated with sodium salt and silkworm pupae oil have lower ERK1/2 phosphorylation (105). The results of this research may help with cardiovascular disease prevention and treatment, maybe through the creation of functional foods or medications.

#### 5.8.8. Silkworm pupae in other important pharmacological functions

Silkworm pupae also have effects that are antigenotoxic (58), antiaging (106), antifatigue (107, 108), and alcohol detoxifying (109) in addition to the pharmacological actions mentioned above. They also suppress the growth of fibroblasts (60). Additionally, silkworm pupae can be used as bioreactors for the expression of complex heterologous proteins, which is essential for the synthesis of recombinant proteins and vaccines (110).

## 6. Impact and usefulness of silkworm during COVID-19 pandemic

In December 2019, the coronavirus epidemic (COVID-19) first appeared. By January 2020, it had extensively spread around the world (111). A novel coronavirus known as severe acute respiratory syndrome coronavirus-2 (SARS-CoV-2) is what causes COVID-19. After the genomic sequence of SARS-CoV-2 was published in January 2020, researchers immediately began to produce detection kits and anti-COVID-19 vaccinations (112–114). To promote the development of these technologies, quick and efficient production of target protein molecules is required. In order to create recombinant proteins, the BEVS (baculovirus expression vector system) was created using the BmNPV (*Bombyx mori* nucleopolyhedrovirus) and silkworm (115, 116). Fetal bovine serum (FBS) is not necessary for the production of recombinant BmNPV when using the silkworm and *E. coli* recombinant bacmid system (117). The BmNPV-silkworm expression method was used to create the SARS-CoV-2 S protein without the utilization of cultured isolates or FBS (118). Japan developed recombinant protein vaccines based on silkworm insect factories that are particularly adopt at producing difficult-to-express vaccine antigens. Thus, silkworm helped a lot to mankind to fight against deadly disease like COVID-19 and the involved mechanism can be used to combat the forecoming deadly diseases.

## 7. Silkworm-based food and medicine safety evaluation

Safety is the primary need a person has for any food or medication. Silkworms are often safer for consumption than other well-known high-protein foods like shrimp and fish (119, 120). Silkworms contain a variety of bioactive substances, each of which serves a unique pharmacological function. However, before evaluating if silkworms can be utilized as food or medicine, the safety of their use must be considered. Concerns about safety are raised with regard to both toxicological safety and allergic reactivity. Transgenic silkworms were used in a 28-day feeding trial on rats to test subacute toxicity. Since the study found no negative reactions or fatalities in rats fed with the transgenic silkworms, it demonstrated that these insects were toxicologically safe, at least for rats to consume (121). The researchers also evaluated the safety of silkworm pupae protein by several acute and subacute toxicological investigations (acute toxicity test; teratogenicity test; and 30-day feeding study). The results indicate that neither fatalities nor abnormalities in histology, clinical chemistry, or hemoglobin levels occurred in any of the experimental groups. Rats may consume up to 1.50 g/kg/d of protein, which is generally regarded as safe (50). Triacylglycerols prevent direct ingestion of the oil, despite its great quality and high amount of unsaturated fatty acids. Structured triacylglycerols produced without the use of solvents are more suitable for direct ingestion, according to a study. Currently, research on allergies to silkworm pupae is common. The allergenicity of silkworm pupae has been examined in numerous research, with the most prevalent allergens being 27-kDa glycoprotein, chitinase precursors, paramyosin, and profilin (15, 122, 123). When consuming silkworm pupae, certain people run the risk of experiencing severe allergic reactions like hives, lightheadedness, irritated skin, and sometimes even shock (15, 119, 124). Allergens should be kept to a bare minimum for food and health products, which can favor using silkworm pupae. Many practical techniques or procedures have currently been investigated to lessen or alleviate the allergenicity of silkworm pupae. According to a study, female silkworm pupae raised on mulberry leaves had lower allergen levels, which may indicate a connection between food and sex in the development of silkworm pupae allergens (125). We can control the production of these allergens in the usage or manufacturing of silkworm pupae, even if we cannot totally avoid their production. Additionally, heat during food processing alters the conformation of the allergen and makes chemical modifications that cause the allergen to lose some of its allergenicity (126, 127). For the pre-treatment of silkworm pupae, hydrolysis and fermentation are viable options since they can successfully lower the allergenicity of foods in the food business (128). According to a recent study, treating protein extracts from silkworm pupae with heat, enzymes, and an acid-base solution greatly decreased their allergenicity. The allergens in the pupae are likely to be affected by high pressure, ultrasound, and microwave treatments as well (129). Overall, silkworms are safe to eat or use as medicine. In spite of the fact that allergic reactions can be triggered by allergens in silkworms, a variety of methods can be used to reduce or destroy them.

## 8. The laws and regulations concerning silkworm as edible insects

To sustain the notion that silkworms are edible insects, a cooperative effort by lawmakers, regulators, and law enforcement is required (130). New EU food legislation has made it feasible to legalize the use of edible insects in Europe. However, it’s critical to consider both the producer’s and the consumer’s safety (131). To encourage the growth and use of these products, more detailed and accurate regulations are needed to monitor the production and consumption of insect food products similar to silkworm larvae and pupae. This could protect customers and highlight the relationship between silkworms and human health and well-being (132).

## 9. Conclusion

The constituents of silkworms, the mechanisms through which they serve as functional diets and therapeutics are emphasized, and the prospects of silkworm pupae as a nutritional supplement and a medication for the treatment of various diseases are shown in this review. The fact that silkworm larvae and pupae are highly nutritious and can be raised in huge quantities swiftly is one of their key advantages (133). They also carry out a number of bioactive processes. This increases the scope for silkworm application in the pharmaceutical and biological sectors. However, the current examination of the pharmaceutical mechanisms of silkworm is insufficient, and many therapeutical experiments are still restricted to *in vitro* and animal studies. Clinical experiments must be conducted instantly to confirm the pharmacological properties of silkworm. The production of nutrient-rich food and pharmaceutical products from silkworms is also limited, and the use of silkworm pupae for human consumption is still very much in its infancy (134). According to the studies we have reviewed, silkworms do have huge potential for use in biomedicine. In order to use silkworm pupae as food and to enhance human health, future research should focus on understanding the pharmacological activities of these organisms, both at the molecular scale and in clinical testing.

## Author contributions

DM, JK, IS, TB, and VD: conceptualization, writing – original draft preparation, and supervision. KP, AN, MG, NN, and SB: preparation of figures and table. DM, JK, VS, HJ, and DJ: conceptualization, preparation of figures, supervision, and review and editing. All authors read and approved the manuscript.
